# In pursuit of off-task thought: mind wandering-performance trade-offs while reading aloud and color naming

**DOI:** 10.3389/fpsyg.2013.00360

**Published:** 2013-06-18

**Authors:** David R. Thomson, Derek Besner, Daniel Smilek

**Affiliations:** Department of Psychology, University of WaterlooWaterloo, ON, Canada

**Keywords:** attention, mind wandering, reading, stroop, performance

## Abstract

The present study investigated whether the frequency of probe-caught mind wandering varied by condition and had any impact on performance in both an item-by-item reading aloud task and a blocked version of the classic Stroop task. Across both experiments, mind wandering rates were found to be quite high and were negatively associated with vocal onset latencies and error rates across conditions. Despite this however, we observed poor correspondence between the effects of task demands on mind wandering rates and the effects of mind wandering on primary task performance. We discuss these findings in relation to attentional resource accounts of mind wandering and suggest that individuals can adjust the relative distribution of executive/attentional resources between internal and external goals in a way that maximizes off-task thought while preserving primary task performance.

The experience of having one's mind unintentionally wander away from the task at hand is ubiquitous and has been the focus of considerable empirical study. Indeed, mind wandering is remarkably prevalent, ranging from around 20% of the time in laboratory reading tasks to around 50% of the time in simple attention tasks (Smallwood et al., 2004) and every day activities (Killingsworth and Gilbert, 2010). In addition, there is a growing body of evidence suggesting that mind wandering can have significant behavioral costs for primary task performance. The present work examines the prevalence and consequences of mind wandering in two well-studied laboratory tasks: reading aloud single words and non-words, and color naming in the context of congruent and incongruent color words (Stroop, 1935). These tasks have been heavily utilized by researchers for decades, and have been used to measure and make inferences about fundamental cognitive processes related to single word reading (as well as other processes such as attention, perception, and memory). However, these tasks have not been considered in the context of mind wandering. We make use of mind wandering in this context in order to inform our understanding of the relation between mind wandering and attention, while at the same time exploring the prevalence and consequences of mind wandering on two as yet unexamined behaviors (i.e., reading aloud and color naming).

Although color naming in the context of a block of incongruent Stroop trials invariably places high demands on attention, specifically selective attention (Bench et al., 1993; Vendrell et al., 1995; see also MacLeod and MacDonald, 2000), the role of attention in word reading is less clear. We therefore begin by considering mind wandering and reading with respect to the concept of attention. The theoretical alternatives relating mind wandering, reading aloud, and attention are shown at the top of Table 1. As can be seen in the table, we consider the possibilities that reading aloud and mind wandering each may or may not require attention and the possibility that if both reading and mind wandering do require attention, the type of attention they require might be the same or different. The bottom of Table 1 shows how the various theoretical combinations would manifest in behavioral outcomes in single word reading and Stroop (in which the task is to actively *avoid* word reading). Specifically, the table shows how the different theoretical accounts would predict (1) the presence or absence of differences in frequencies of mind wandering across different reading conditions that require different amounts of attention (e.g., while reading words vs. non-words or while color naming on congruent vs. incongruent trials) and (2) the extent to which bouts of mind wandering would influence performance (reaction time and accuracy) in reading aloud and the Stroop task. The central point to take away here, is that regardless of the theoretical relationships between reading/naming aloud, attention, and mind wandering—variations in mind wandering rates across conditions and mind wandering-related performance costs should either both be present or both be absent. Assessing these alternatives is the purpose of the present work.

**Table 1:** **A representation of the theoretical relations between attention, reading and mind wandering, and the empirical predictions that follow from these alternatives with regards to whether differences in mind wandering across condition should be observed and whether mind wandering-related performance deficits should be observed**.

	**1**	**2**	**3**	**4**	**5**
**THEORETICAL ALTERNATIVES**					
Mind wandering requires attention	Yes	Yes	Yes	No	No
Reading requires attention	Yes	Yes	No	Yes	No
Reading and mind wandering require the same type of attention	Yes	No	–	–	–
**EMPIRICAL PREDICTIONS**					
Differences in mind wandering across conditions within task	Yes	No	No	No	No
Mind wandering-related performance deficits	Yes	No	No	No	No

In what follows we review various theoretical positions outlined in Table 1. First we consider existing views on the relation between mind wandering and attention. We then briefly review the current controversy regarding the involvement of attention in reading aloud. Finally, we discuss the possibility that mind wandering and reading aloud might require the same attentional resources.

## Mind wandering and attention

Smallwood and Schooler (2006) propose an account of mind wandering in which controlled processing directs attention away from external stimuli and goals toward internal, personally relevant, thoughts and goals. This account suggests that the amount of mind wandering that occurs in a given task context is directly related to the executive and attentional demands of the primary task, since the act of mind wandering itself requires that at least some executive resources be available to direct the focus of attention internally. Specifically, it is argued that the more controlled processing that is required by a primary task, the lower the incidence of mind wandering will be (Smallwood and Schooler, 2006; Smallwood, 2010). It should be noted that this type of relationship has also been posited to explain dual-task performance, whereby mental resources must be divided among two distinct tasks (Pashler, 1994) perhaps according to some principle that maximizes performance on both tasks (Navon and Gopher, 1979). This distribution process is likely to hinder task performance, particularly when one or more of the tasks places high demands on executive resources (Moscovitch, 1994). Theoretical accounts of resource distribution in mind wandering may therefore mirror those posited to explain dual-task interference in prior work. Indeed, some evidence does exist for this contention. For example, high perceptual load has been shown to decrease reports of off-task thought relative to low perceptual load in a visual search task (Forster and Lavie, 2009). In addition, more demanding go/no-go tasks (with a low target probability) result in lower mind wandering rates than easier (high target probability) versions of the same task (Smallwood et al., 2007). Finally, it has been shown that mind wandering rates are significantly higher in the second half of a sustained attention-to-response task (SART, see Robertson et al., 1997) relative to the first half, presumably because the task demands decrease as experience with the task increases (Smallwood et al., 2004). Therefore, in at least some contexts, task demands predict the frequency of mind wandering.

The relative frequencies of mind wandering across tasks (and across conditions within tasks) aside, the most important issue concerning research on mind wandering is the way in which off-task thought hinders or disrupts primary task performance. It has been shown for example that SART errors are related to absentmindedness assessed via questionnaire (Manly et al., 1999) and that faster responding in the SART task occurs in blocks in which mind wandering is shown to occur (Smallwood et al., 2004). In addition, individuals display increased response variability in the trials prior to off-task reports relative to on-task reports in a continuous (metronome) response task (Seli et al., 2013). It therefore seems as though there is a strong correspondence between the executive processes required for mind wandering and those required for sustained attention tasks requiring overt manual responding.

It is therefore apparent that there is a wealth of empirical evidence indicating that mind wandering does not typify a lack of attention, but rather, a re-direction of attention away from the external environment. Returning to Table 1, it seems as though theoretical alternatives four and five can largely be ruled out. Given that mind wandering is believed to require attention, options one, two, and three remain as possible theoretical alternatives regarding the relationship between mind wandering, reading at the single word level, and attention. Mainly, whether or not reading requires attention, and if so, whether it requires the same type of attention that is involved in mind wandering.

## Reading and attention

For skilled readers, the translation of print into speech seems effortless, and is widely considered to be unavoidable, as in the classic Stroop task [1935; see also MacLeod's (1991) review]. Indeed, this phenomenology has been formalized in theoretical accounts in which phonological re-coding of print is widely argued to reflect “automatic” processes that occur without the need for any kind of “attention” (e.g., LaBerge and Samuels, 1974; Posner and Snyder, 1975; Neely and Kahan, 2001; Brown et al., 2002 among many others). For example, LaBerge and Samuels (1974) argue that: “With enough practice, of course, activation of the stimulus code excites the response code without attentional assistance” (p. 316). And Brown et al. (2002) have contended that: “[well-known words] can activate their lexical representations … without having visual attention focused on them” (p. 237). In contrast, other researchers have argued that several different kinds of attention play a prominent role when identifying words in a variety of paradigms. For example, spatial attention has been implicated in word reading in the context of the Stroop paradigm (Besner and Stolz, 1999; Roberts and Besner, 2005), as well as in the context of the spatial cueing paradigm (McCann et al., 1992; Lachter et al., 2004; Waechter et al., 2011). Moreover, it has been argued that more spatial attention is required for sublexical than lexical processing (Waechter et al., 2012), and that executive processes (intention) can play a role in the context of the Task Set paradigm when reading both words and non-words aloud (Besner and Care, 2003; O'Malley and Besner, 2012). Thus, although many specific types of attention have been implicated in word identification, perhaps the most relevant for the present study is the role of central attention, as it is arguably synonymous with the type of central executive resources implicated in mind wandering.

Central attention has been implicated in the process of translating orthographic representations into speech (reading aloud). For example, it has been argued that more central attention is required in the context of sublexical phonology than lexical processing when reading aloud in the Psychological Refractory Period (PRP) paradigm (Reynolds and Besner, 2006; O'Malley et al., 2008). Therefore, although there remains considerable debate in the literature regarding whether processes related to reading require attention or whether such processes occur “automatically,” those researchers who do suggest a prominent role of attention suggest that the act of reading aloud depends on central attention. Returning to Table 1, the first three alternatives still seem to be plausible, however the evidence points to the conclusion that at least some executive resources (or central attention) are required for reading aloud, suggesting that options one and two are the most plausible. Given this, the incidence and consequences of mind wandering on reading aloud would seem to depend on whether mind wandering and reading require the same *type* of attention.

## Do mind wandering and reading share the same type of attention?

It is often assumed that if two tasks rely on the same kind of attention (i.e., they make use of the same executive resources) then performing both tasks simultaneously ought to cause interference (deficits in task performance). Indeed, this idea has been borne out in a host of empirical demonstrations. For example, it has been shown that one's ability to perform a visual search task is hindered by the additional requirement to maintain a memory load (Schneider and Shiffrin, 1977), one's ability to drive a car is affected by use of a cellular phone (Strayer and Johnston, 2001), and one's ability to learn novel categories is disrupted by a working memory task (Zeithamova and Maddox, 2006). It therefore stands to reason that if mind wandering and reading rely on the same attentional resources, we should expect to see two things: (1) as the amount of resources required by the primary reading task increases, mind wandering rates should decrease, and (2) devoting resources to mind wandering should result in deficits in reading performance.

While no prior work has assessed the relationship between mind wandering and reading aloud single words and non-words, there has been considerable recent interest concerning mind wandering and reading in general. For example, Feng et al. (2013) found that participants mind wandered *more* while reading difficult compared to easy texts (in contrast to the findings from sustained attention tasks) and that mind wandering had a greater negative impact on the difficult relative to easy texts. In contrast, Unsworth and McMillan (2012) found that topic interest and motivation better accounted for mind wandering differences across texts that varied in difficulty. It is therefore an open question as to whether mind wandering rates will vary as a function of the demands placed on controlled processing in tasks that involve reading and overt vocal responses in an item-by-item paradigm. However, general performance deficits owing to mind wandering while reading (silently) have been well documented. For example research on mindless reading has revealed that mind wandering impairs participants' ability to understand a complex narrative (Smallwood et al., 2008b). In addition, mindless reading also appears to be associated with longer fixation durations to individual words (Reichle et al., 2010) and an increase in eye blink rates relative to normal reading (Smilek et al., 2010), and the speeding of manual key-presses that advance text in a word-by-word reading paradigm (Franklin et al., 2011).

There is therefore, a large body of evidence suggesting that reading likely shares attentional resources with mind wandering since these behaviors seem to interfere with one another. Consequently, the available evidence seems to suggest that of our theoretical alternatives outlined in Table 1, the first option is the most probable. Given this, we would expect that mind wandering rates should vary as a function of task demands when reading aloud and color naming, *and* that mind wandering should affect reading performance in some way.

## The present studies

In the two experiments presented here, we evaluate reading performance directly by having participants read aloud words and non-words (Experiment 1) and indirectly by measuring color naming in the Stroop task (Experiment 2). In both experiments we varied possible attentional demands of the task by varying either word familiarity (Experiment 1: low and high frequency words and non-words) or word-color congruity (Experiment 2: congruent vs. incongruent). In both experiments, we included intermittent thought probes (Smallwood and Schooler, 2006; Smallwood et al., 2007) asking participants to report whether they were on or off task at the moment just prior to the probe. In this way it was possible to evaluate the behavioral outcomes shown in Table 1; namely, (1) whether the frequency of mind wandering varied as a function of the different demands placed on attention in different conditions and (2) whether mind wandering had an effect on performance (by comparing performance just prior to on and off-task reports).

To foreshadow the results, we find that, in both of the experiments reported here, mind wandering rates vary systematically as a function of the demands placed on controlled attention within each task, but surprisingly, this does not correspond with mind wandering-related deficits in performance. Importantly, this striking pattern of results is different from any of the predicted outcomes outlined in Table 1. According to the theoretical possibilities outlined in Table 1, variations in mind wandering rates across conditions and performance related deficits owing to mind wandering ought to co-occur. Our pattern of findings suggests that a different conceptual framework is needed to understand the interplay between mind wandering, reading and attention; a conceptual framework that we develop in the General Discussion.

## Experiment 1

The purpose of this experiment was to examine whether mind-wandering rates vary as a function of item familiarity and/or lexical status (word or non-word) in a simple item-by-item reading aloud task. It is well known that more familiar words (high frequency ones) are read aloud faster than less familiar words (low frequency ones) and that words are typically read faster than non-words (e.g., Forster and Chambers, 1973; Frederiksen and Kroll, 1976). We therefore had participants read high and low frequency words and non-words in separate blocks of trials while also assessing mind wandering via pseudo-randomly presented thought probes. Given the view that reading non-words aloud is more attentionally demanding than reading words aloud (e.g., Reynolds and Besner, 2006; O'Malley et al., 2008; O'Malley and Besner, 2012; Waechter et al., 2012), we expected that reading non-words aloud should yield less mind wandering than do words under an executive resources view of mind wandering. Importantly, we also examined whether there are any measurable differences in reading aloud performance while mind wandering relative to when subjects report being focused on-task.

### Method

#### Participants

This experiment was conducted on two independent samples of participants (run in two separate academic terms) in order to test the stability of any observed differences in mind wandering across conditions, and also to increase our power to detect any performance costs associated with mind wandering. All participants were undergraduates from the University of Waterloo, who participated in exchange for course credit. Only participants who reported English as their primary written and oral language were recruited. Sample 1 consisted of 36 participants (12 males, 24 females) with a mean age of 19.5 years. Sample 2 also consisted of 36 participants (9 males, 27 females) with a mean age of 20.0 years.

#### Apparatus and stimuli

Stimuli were presented using a MacBook Pro computer with a 2.26 GHz Intel Core 2 Duo processor connected to an LG Flatron 21.5 inch LCD monitor. Participants' vocal responses were detected using the computer's built-in microphone; key press responses were collected from a separate keyboard connected to the computer. The experiment was programmed in Python Version 2.6.6 (www.python.org/) and run using PsychoPy software (Peirce, 2007). Stimuli consisted of 200 words with a Kucera and Francis (1967) (KF) mean frequency of 329.5 (*SD* = 534.9) (henceforth referred to as high frequency words), 200 words with a mean KF frequency of 6.3 (*SD* = 5.7) (henceforth referred to as low frequency words), and 200 pronounceable non-words. High and low frequency words were obtained using the Washington University Speech and Hearing Lab Neighborhood Database (128.252.27.56/Neighborhood/Home.asp) with the constraints that all words be five letters in length. Non-words were generated using the ARC non-word database (Rastle et al., 2002) with the constraints that all letter strings were five letters long, consisted of legal bigrams, orthographically existing onsets, and a minimum orthographic neighborhood size of 1. High frequency, low frequency, and non-words had orthographic neighborhood sizes of 3.57, 3.40, and 3.66 respectively (as measured by Coltheart's N, taken from: MCWord: An orthographic wordform database, www.neuro.mcw.edu) and did not significantly differ from one another, *F*_(2, 597)_ = 0.52, *p* = 0.59. All stimuli were presented in white Arial lowercase font on a gray background and subtended a visual angle of approximately 1.0° vertically and 3.5° degrees horizontally. The items are available on request.

### Procedure

Participants were told that they would view a single letter string at a time on the computer screen, and that their task was to read each item aloud. The three item types (high frequency, low frequency and non-words) were presented in a blocked manner, resulting in three blocks of 200 trials each, for a total of 600 experimental trials. Block-order was counter-balanced across participants. Vocal onsets were detected by the computer at which point the stimulus was replaced by a central fixation cross that subtended 1° of visual angle. The experimenter then coded whether the trial was “correct,” “incorrect,” or “spoiled” by use of the keyboard, after which the fixation cross remained on the screen for an additional 500 ms. An utterance was considered to be incorrect if an erroneous phoneme was produced, or if an orthographic or phonological neighbor was produced instead of the target stimulus. One participant from sample 1 was excluded from all analyses on the basis of an exceptionally high percentage of errors in the non-word condition (31%); an additional participant was tested to complete the counterbalance. A trial was considered to be “spoiled” if something other than the participant's voice triggered the microphone, or if the microphone failed to be triggered by the participant's voice, which accounted for an average of 1.8% of trials (there was no relation between the frequency of spoiled trials and the frequency of mind wandering among the individuals tested).

In addition to the primary reading task, participants were informed that at various points throughout the experiment their thoughts would be probed. These thought probes were signaled by a pure tone (350 ms in duration), at which point participants were asked to indicate whether, to the best of their knowledge, their attention was focused on or off the reading task. Prior to the experiment, participants were provided with the following definition of on-task vs. off-task thought (mind wandering), taken from Smallwood et al. (2007): “During this experiment you will be asked at various points whether your attention is firmly directed toward the task, or alternatively you may be aware of other things than just the task. Occasionally you may find as you are reading that you begin thinking about something completely unrelated to what you are reading; this is what we refer to as mind wandering.” Participants were also provided with written definitions of on- and off-task thought that they were free to consult throughout the experiment. These written definitions were stated as follows: “On-task: Just prior to the tone, your attention was firmly directed toward the task. Off-task: Just prior to the tone, you were aware of things other than the task; you were thinking of something completely unrelated to what you were reading.” Participants indicated that they were on-task by pressing the “z” key on the keyboard and indicated that they were off-task by pressing the “m” key (this assignment was reversed for half of the participants). For each block of trials (high frequency, low frequency and non-words) there were ten thought probes. Thought probes were presented pseudo-randomly throughout each block with the constraints that no probes were encountered within the first 15 trials of each block and that probes were separated by a minimum of 12 intervening reading trials. The experiment took approximately 25 minutes to complete.

### Results

#### Mind wandering rates

**Figure 1:**
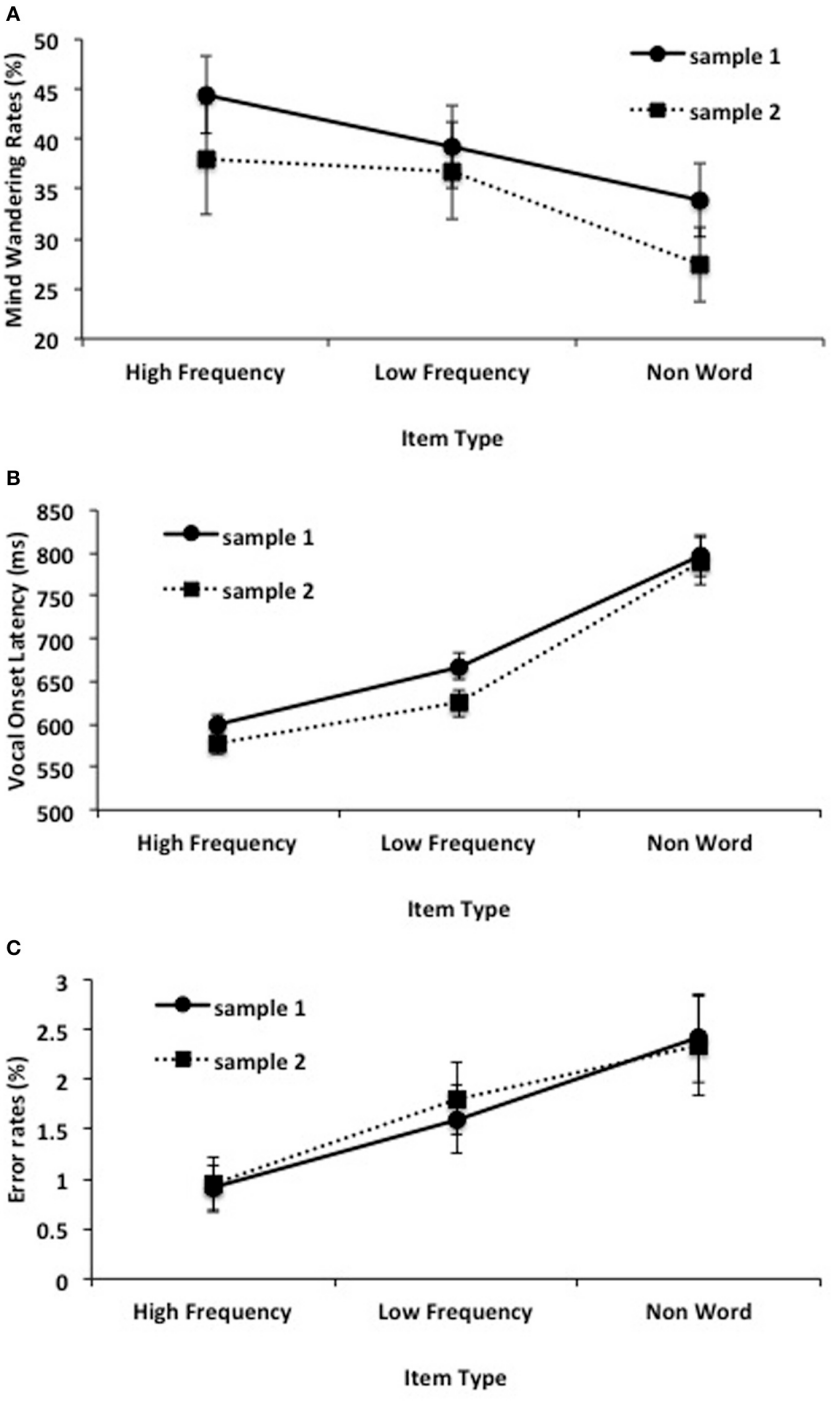
**(A)** Mean percentages of “off-task” responses to the thought probes as a function of item type and sample. **(B)** Mean vocal onset latencies for reading aloud as a function of item type and sample, and **(C)** corresponding error percentages. Error bars represent one standard error of the mean.

The mean frequency of off-task reports collapsed across item type and sample was 37.1%. Frequencies of off-task reports for each participant were submitted to an ANOVA that treated item type (high frequency, low frequency, non-word) as a within subject factor, and sample (1, 2) as a between subjects factor. There was a significant effect of item type, *F*_(2, 140)_ = 5.20, *p* = 0.007, η^2^_*p*_ = 0.07, but no main effect of sample, nor was there a sample x item type interaction, (*F*'s < 1). Follow-up paired comparisons (collapsed across sample) revealed that mind wandering rates were higher for high frequency words (41.5%) than non-words (31.1%), *t*_(71)_ = 3.05, *p* = 0.003, *d* = 0.36, and higher for low frequency words (38.8%) than non-words, *t*_(71)_ = 2.34, *p* = 0.022, *d* = 0.28. Mind wandering rates did not significantly differ between high and low frequency words, *t* < 1. Mean mind wandering rates as a function of item type and sample are depicted in Figure 1A.

#### Response times and percentage errors

Correct response times (RTs) for the reading task were first submitted to a non-recursive outlier procedure that eliminates observations using a standard deviation cut-off based on cell size (Van Selst and Jolicoeur, 1994). This procedure resulted in the exclusion of an average of 3.2% of the observations within each cell. Paired comparisons revealed that more RTs were excluded in the low frequency condition (3.21%) relative to the non-word condition (3.06%), *t*_(71)_ = 3.06, *p* = 0.003, *d* = 0.36, there were no other significant differences in the percentage of outliers excluded. The remaining correct mean RTs for each participant in each condition were then submitted to an ANOVA that treated item type (high frequency, low frequency, non-word) as a within subject factor and sample (1, 2) as a between subjects factor. This analysis revealed a main effect of item type, *F*_(2, 140)_ = 88.60, *p* < 0.001, η^2^_*p*_ = 0.56, but no main effect of sample, nor was there a sample x item type interaction, (*F*'s < 1). Follow-up comparisons (collapsed across sample) revealed mean RTs to be significantly faster in the high frequency condition (589 ms) than in either the low frequency condition (646 ms), *t*_(71)_ = 8.53, *p* < 0.001, *d* = 1.01, or the non-word condition (793 ms), *t*_(71)_ = 10.57, *p* < 0.001, *d* = 1.25. RTs in the low frequency condition were significantly faster than in the non-word condition, *t*_(71)_ = 8.10, *p* < 0.001, *d* = 0.95; a replication of the well-known effect of item frequency and lexical status on reading times first reported by Forster and Chambers (1973).

We also conducted an item analysis by submitting mean RTs collapsed across subjects to a one-way ANOVA with item type as a between condition factor. This revealed a significant effect of item type, *F*_(2, 597)_ = 726.53, *p* < 0.001, η^2^ = 0.71. Mean RTs as a function of item type and sample are depicted in Figure 1B.

The percentages of errors committed as a function of item type for each participant were also submitted to an ANOVA with item type (high frequency, low frequency, non-word) as a within subject factor and sample (1, 2) as a between subject factor. There was a significant effect of item type, *F*_(2, 140)_ = 19.04, *p* < 0.001, η^2^_*p*_ = 0.21. There was no main effect of sample nor was there a sample x item type interaction, *F*'s < 1. Follow up comparisons (collapsed across sample) revealed significantly fewer errors in the high frequency condition (0.9%) than in the low frequency condition (1.7%), *t*_(71)_ = 4.79, *p* < 0.001, *d* = 0.56, or non-word condition (2.4%), *t*_(71)_ = 5.30, *p* < 0.001, *d* = 0.62. In addition, fewer errors were committed in the low frequency condition relative to the non-word condition, *t*_(71)_ = 2.69, *p* = 0.009, *d* = 0.32. Mean error percentages as a function of item type and sample are depicted in Figure 1C.

#### Performance by probe response

In order to assess whether off-task reports were accompanied by any consequences on reading performance, mean correct RTs were computed from the five trials prior to each thought probe as a function of whether the proceeding thought probe received an “on-task” or “off-task” response^1^. Prior work from our lab has shown that the five trials prior to a thought probe provide reliable measures of performance differences as a function of probe response in other tasks such as synchronously responding to a metronome (Seli et al., 2013). On-task and off-task RTs as a function of item type (high frequency, low frequency, non-word) were submitted to a repeated measures ANOVA. Since mean RTs were compared within participant, only participants with at least 1 “on-task” and at least 1 “off-task” response for each item type were included in the analysis. This analysis revealed a main effect of item type, *F*_(2, 108)_ = 33.27, *p* < 0.001, η^2^_*p*_ = 0.38; analogous to the effects of item frequency and lexical status seen in the overall pattern of RTs. There was however no main effect of probe response, with RTs being similar on the five trials prior to both on-task (693 ms) and off-task (685 ms) reports. Importantly, there was no item type x probe response interaction (*F* < 1), indicating that task difficulty did not modulate or dictate performance differences on the trials preceding on and off-task reports.

We also assessed corresponding error percentages on the five trials prior to the thought probes and submitted them to a 3 (item type) × 2 (probe response) ANOVA. This analysis yielded no significant effects involving probe response (*F*'s < 1), in-line with the pattern of RTs observed.

### Discussion

The primary purpose of Experiment 1 was twofold: (1) to assess the prevalence of mind wandering in a standard item by item reading aloud paradigm as a function of the demands of the reading task (lexical vs. sublexical processing), and (2) to assess whether mind wandering affected performance on the primary task. In line with the predictions of attentional resource views of mind wandering (i.e., Smallwood and Schooler, 2006; Smallwood, 2010), the condition with the slowest RT and highest error rate (the non-word condition) was also the condition in which the lowest frequency of mind wandering was observed. This variation in mind wandering across conditions suggests that reading aloud is affected by attention (likely the same type of attention that is involved in mind wandering). Importantly however, there were no effects of mind wandering on reading performance itself. That is, mind wandering did not affect either RT or errors when reading aloud.

The results of Experiment 1 are surprising. It was expected that if mind wandering rates varied by condition (lexical vs. sublexical processing) then mind wandering-related deficits in performance should also have occurred, since the former result suggests that mind wandering and reading aloud require the same type of attention. The failure to see any performance costs when reading aloud suggests that the predictions outlined in Table 1 are too simplistic. It seems necessary to posit some way in which primary task demands can affect the frequency of reported mind wandering, yet mind wandering itself does not affect performance on the primary task. Specifically, we suggest that participants may modulate the degree of mind wandering they engage in, in-line with the primary task demands, so that the executive/attentional resources devoted to mind wandering do not impinge on those required for primary task performance. We return to this point in the General Discussion. Before speculating on such processes however, we sought to determine whether the pattern of results observed in Experiment 1 would also manifest in yet another well-studied item-by-item laboratory paradigm involving overt vocal responding, namely, the Stroop task.

## Experiment 2

The primary purpose of Experiment 2 is to examine the potential influence of mind wandering on perhaps the most robust, well-studied behavioral effect in cognitive psychology; Stroop [1935; see MacLeod (1991) for a review]. Given that variations in mind wandering across conditions in Experiment 1 did not co-occur with mind wandering-related performance deficits (as was predicted) we seek to provide a replication of this surprising finding in Experiment 2. In a standard Stroop task, the reader names the print color of a color word that either matches or mismatches the print color of that word. This Stroop effect can be measured by the difference in RT to incongruent items (i.e., the word “red” in green) and congruent items (i.e., the word “red” in red). Stroop-type tasks have been relied upon by researchers to measure and explore the processes related to reading (Brown et al., 2002), attention (Besner and Stolz, 1999; Roberts and Besner, 2005), as well as learning and automaticity (Crump et al., 2006; Milliken et al., 2012), to name a few.

The Stroop task lends itself well to the present study, since the executive resources required to accurately name the color of congruent and incongruent trials differ markedly, with incongruent trials placing greater demands on attention and controlled processing, by, for example, necessitating both goal maintenance and competition resolution from competing stimulus dimensions (Kane and Engle, 2003); executive processes not required for performance on congruent trials. We therefore expected mind wandering rates to be significantly higher during a block of incongruent Stroop trials relative to a block of congruent trials. If so, the question then is whether the dependence of task demands on mind wandering rates co-occurs with mind wandering-related performance deficits or if the pattern of results observed with reading aloud in Experiment 1 can be extended to naming aloud as well.

### Method

#### Participants

Forty undergraduates (9 males, 31 females) with a mean age of 20.3 years from the University of Waterloo participated in exchange for course credit. All participants reported normal color vision and English as their primary written and oral language.

#### Apparatus and stimuli

Stimuli were presented using an iMac computer with a 2.26 GHz Intel Core 2 Duo processor and a 21.5 inch LCD monitor. Participants' vocal responses were detected using the computer's built-in microphone; key press responses were collected from a separate keyboard connected to the computer. All stimuli were presented in Arial lowercase font on a black background and subtended a visual angle of approximately 1.0° vertically and 3.5° horizontally. The items the words “red,” “green,” “blue,” and “yellow,” and were presented in the colors red, green, blue, and yellow. Congruent trials were created by presenting words in their matching ink colors (i.e., the word “red” in red) and incongruent trials were created by presenting words in a mismatching ink color (i.e., the word “green” in blue, red, or yellow).

#### Procedure

Participants were told that they would view a single color word at a time on the computer screen, and that their task was to name aloud the color that the word was presented in and to ignore the word itself. The two item types (congruent and incongruent) were blocked, with block-order counter-balanced across participants. Note that we chose not to include neutral trials in the present experiment, this was done because, (1) any differences in attentional demands between congruent and neutral trials is likely minimal, and (2) we did not want to reduce the block size for congruent and incongruent trial types so as to have sufficient power to observe mind wandering differences across conditions as well as any potential performance costs associated with on and off-task reports. The experiment was run in blocks of 150 trials (two congruent blocks and two incongruent blocks, interleaved). Vocal onsets were detected by the computer at which point the stimulus was replaced by a central fixation cross that subtended 1° of visual angle. The experimenter then coded whether the trial was “correct,” “incorrect,” or “spoiled” by use of the keyboard, after which the fixation cross remained on the screen for an additional 500 ms. An utterance was considered to be incorrect if a color name other than the color of the target stimulus was uttered, or if the participant began uttering an incorrect color name and then self-corrected (i.e., “bl—yellow”). A trial was considered to be “spoiled” if something other than the participant's voice triggered the microphone, or if the microphone failed to be triggered by the participant's voice, which accounted for an average of 2.0% of trials (there was no relation between the frequency of spoiled trials and the frequency of mind wandering among the individuals tested).

In addition to the primary Stroop task, participants were pseudo-randomly asked to indicate whether their thoughts were most recently focused on or off the task in the same manner as in Experiment 1. Six thought probes were presented within each block, for a total of 12 thought probes for each item type (congruent vs. incongruent). The experiment took approximately 30 min to complete.

### Results

#### Mind wandering rates

**Figure 2:**
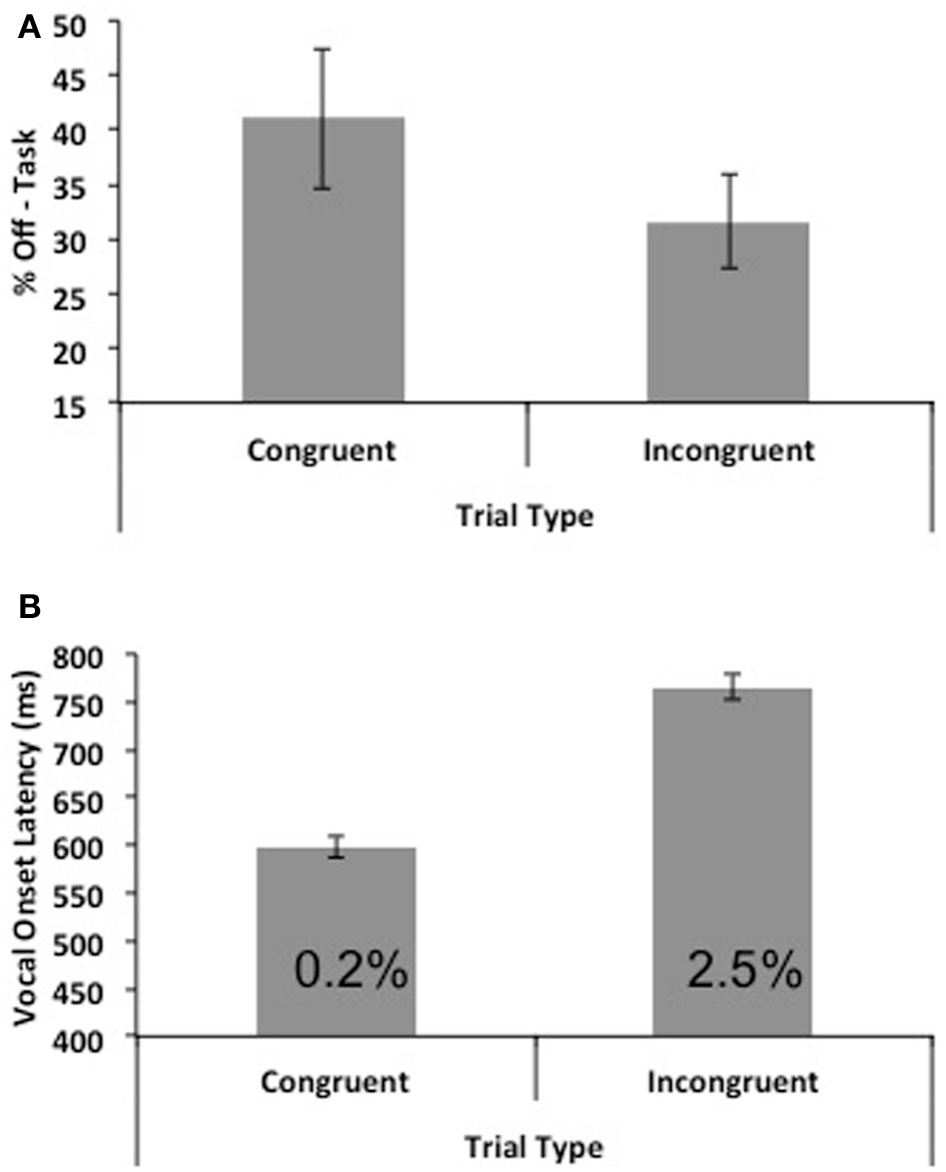
**(A)** Mean percentages of “off-task” responses to the thought probes as a function of trial type. **(B)** Mean vocal onset latencies as a function of trial type and sample (and corresponding error percentages). Error bars represent one standard error of the mean.

The mean frequency of off-task reports collapsed across trial type was 36%. Frequencies of off-task reports for each participant were submitted to a paired samples *t*-test that compared the frequency of off-task reports between congruent and incongruent blocks. There was a significant effect of trial type on off-task reports, *t*_(39)_ = 2.14, *p* = 0.039, *d* = 0.34, with a lower frequency of off-task reports occurring for incongruent trials (31%) than for congruent trials (41%). Mean mind wandering rates as a function of trial type are depicted in Figure 2A.

#### Response times and percentage errors

Correct RTs as a function of trial type were submitted to the same outlier elimination procedure as in Experiment 1. This resulted in the exclusion of 2.6% of RTs from further analysis. There was no difference in the percentage of outliers removed between the congruent and incongruent conditions. Mean RTs for each participant were then submitted to a paired-samples *t*-test, that revealed congruent RTs (599 ms) to be significantly faster than incongruent RTs (765 ms), *t*_(39)_ = 15.96, *p* < 0.001, *d* = 2.52; the well-known Stroop effect. In addition, mean percentage errors were also lower for congruent trials (0.2%) relative to incongruent trials (2.5%), *t*_(39)_ = 3.21, *p* = 0.003, *d* = 0.51, ruling out a speed-accuracy trade-off interpretation of the pattern of RTs. Mean RTs and corresponding error rates are depicted in Figure 2B.

#### Performance by probe response

As in Experiment 1, mean correct RTs for the five trials prior to each thought probe were submitted to a repeated measures ANOVA that included trial type (congruent, incongruent) and probe response (“on-task”/“off-task”) as factors^2^. There was a main effect of trial type, *F*_(1, 25)_ = 72.63, *p* < 0.001, η^2^_*p*_ = 0.74, with RTs being faster on congruent (577 ms) relative to incongruent (760 ms) trials; a replication of the pattern of RTs seen in the overall analysis of the Stroop effect. In contrast, there was no main effect of probe response, with similar RTs prior to on-task (686 ms) relative to off-task (699 ms) probe responses. In addition, there was no trial type x probe response interaction.

We also computed corresponding percentage errors on the five trials prior to the thought probes and submitted them to a repeated measures ANOVA that included trial type and probe response as factors. This analysis revealed a significant main effect of probe response on errors, *F*_(1, 25)_ = 4.47, *p* = 0.045, η^2^_*p*_ = 0.15, with errors tending to be higher prior to “off-task” relative to “on-task” responses (1.6 and 0.5% respectively). There was no trial type x probe response interaction.

### Discussion

Similar to the results of Experiment 1 and consistent with attentional resource accounts of mind wandering, the results of Experiment 2 again showed that the condition with the slowest RT and highest error rate (the incongruent condition) was the condition with the lowest frequency of reported mind wandering. This again suggests that the attentional processes related to reading (or the prevention of reading in the incongruent condition) overlap with the attentional processes related to mind wandering. Crucially though, we again found very poor correspondence between variations in mind wandering across conditions and performance deficits related to mind wandering (no difference in the pattern of RTs and a small difference in the pattern of errors), counter to the empirical predictions outlined in the introduction and in Table 1. The implications of these results, together with those of Experiment 1 are taken up in the General Discussion.

## General discussion

The primary purpose of the present study was to assess the prevalence of mind wandering in a reading aloud task and a Stroop task and to determine whether (a) the incidence of mind wandering varies as a function of the conditions in these experiments and (b) if so, whether there are any consequences of mind wandering on performance. As evidenced in Table 1, we reasoned that if reading and mind wandering both require attention (as prior work has indicated) and if they rely on the same type of attention, then variations in mind wandering as a function of task demands should co-occur with mind wandering-related performance deficits. The results were clear: the frequency of off-task reports was quite high across both experiments (almost 40% on average) and varied across conditions with mind wandering rates decreasing as RT and error rates increased. Importantly though, despite the clear effects of task demands on mind wandering rates (suggesting that mind wandering relies on the same resources as the tasks studied here), no consistent effects of mind wandering on primary task performance were observed. In other words, the effects of the primary task on mind wandering do not predict the effects of mind wandering on the primary task, a finding that has important ramifications for theoretical accounts of mind wandering.

The lack of performance costs associated with mind wandering in the present experiments is particularly striking given the number of studies that have demonstrated deficits in performance during mind wandering episodes in other tasks and contexts (Smallwood et al., 2004, 2008a, b; Reichle et al., 2010; Franklin et al., 2011; Seli et al., 2013). These deficits are argued to occur because executive resources are directed away from external task-related goals and information toward internal task-unrelated goals and thoughts (Smallwood and Schooler, 2006). This “perceptual decoupling” is argued to result in reduced processing of external sensory information (Smallwood et al., 2007, 2008a; Schooler et al., 2011; Smallwood, 2011) that results in changes in overt behavior (i.e., such as fixation durations and blink rates) as well as deficits in objective task performance. Importantly, the present experiments are the first to assess potential influences of mind wandering on both reading words and non-words aloud and color naming in the face of competing words. The scarcity of performance costs associated with mind wandering seen here is surprising, given the theoretical predictions outlined in the introduction and seen in Table 1. At least two possible interpretations of these surprising findings are suggested, which are outlined below.

The lack of correspondence between variations in mind wandering and performance costs owing to mind wandering in the present study may mean that mind wandering relies upon attentional resources that are wholly distinct from the attentional resources associated with phonological re-coding and color naming. This strikes us as an unlikely proposition given the fact that probe-caught mind wandering rates, both in the present experiments as well as in prior work (Smallwood et al., 2007; Forster and Lavie, 2009), vary systematically with the objective demands of the primary task. In other words, if mind wandering and task performance result from qualitatively different executive/attentional resources, then increased recruitment of one type of resource should not co-occur with a decreased recruitment of the other. Specifically, to-date, resource competition has been argued to be a central aspect of the relationship between mind wandering and primary task performance (Smallwood, 2010).

**Figure 3:**
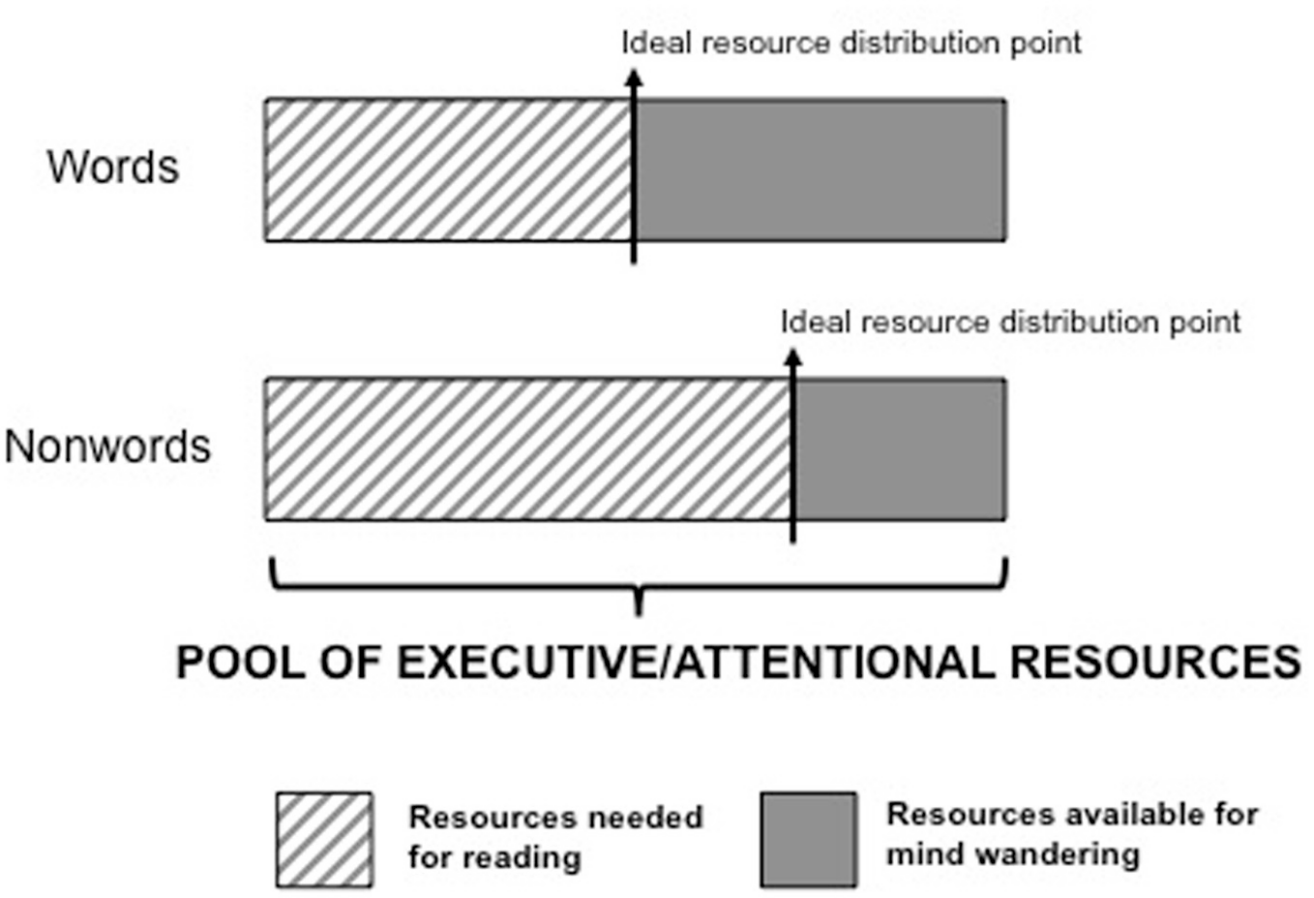
**A theoretical depiction of the distribution of executive/attentional resources in the word reading and non-word reading conditions of Experiment 1**. The ideal distribution point is shown; the point at which mind wandering is maximized without impinging on resources needed for reading. A distribution point to the left of the one shown would result in primary task performance costs, whereas a distribution point to the right of the one shown would result in a decrease in mind wandering with no appreciable benefits to primary task performance.

A different account of the results reported here is that mind wandering and overt reading aloud and color naming *do* rely on the same attentional resources. In other words, returning to Table 1, the first theoretical alternative may be the most accurate. In order for this to be the case, and yet for variations in mind wandering rates and performance deficits to *not* co-occur (as the Table would predict), a novel conceptualization of the relationship between mind wandering, attention, and task performance is required. In short, (1) the lack of any measurable costs to performance as a function of mind wandering seen here suggests that reading aloud (or the prevention of reading in the Stroop task) requires attention, but that not all attentional resources are required in order to achieve a high level of performance. (2) We speculate that for some tasks, there is a “saturation” point beyond which additional resource investment proves ineffective at improving performance. (3) “Unused” attentional resources are then devoted to mind wandering (thus accounting for the robust variation in mind wandering rates across conditions in the work reported here). (4) Individuals are able to “modulate” the amount of off-task thought they engage in so as to devote the maximum amount of “unused” resources toward the pursuit of internal thoughts and goals, without impinging on the resources needed for the primary task (and thus not interfering with task performance). An illustration of this theoretical process is shown in Figure 3. Finally, (5) the ease with which an individual can appropriately distribute executive resources may depend on the extent to which one can “monitor” primary task performance in an online manner^3^; tasks that afford little feedback will be particularly ill-suited for this process and so mind wandering is likely to consume resources needed for the primary task, thus resulting in mind wandering-related performance deficits in such tasks.

Applying the above logic to prior work, perhaps the reason performance costs are associated with mind wandering in other attention and reading tasks (i.e., SART, reading comprehension) is because the resource distribution process fails due to insufficient feedback (or performance monitoring). In the SART task for example, the relatively low target probabilities afford little opportunity for individuals to adjust off-task thoughts compared with the present work, in which performance can be assessed on every trial. Likewise, when reading a passage of text, only when one has failed to comprehend the main point of the passage, does one realize that off-task thought was too frequent and that important premises were missed, at which point it is too late to re-distribute resources accordingly. This “distribution” account of mind wandering would therefore predict that as feedback on task performance increases, costs associated with mind wandering decrease. While speculative at present, the idea that internal/external resource distribution trade-offs depend on one's ability to monitor online performance suggests an interesting avenue for future study. This theoretical proposal also predicts that in particularly difficult tasks, in which all executive resources are needed for good performance, any mind wandering observed will interfere with performance on the primary task; an issue to be pursued in future studies.

Finally, the present findings may suggest a potentially useful way in which researchers can make use of mind wandering rates in future research. Specifically, because mind wandering rates proved to be reliable predictors of RT and error rates in both of the experiments reported here, yet probe response (on or off-task) did not interact with the primary behavioral measure of interest (word frequency effect, Stroop effect) the frequency of mind wandering rates across tasks and across conditions within tasks may serve as an independent diagnostic test of the relative demands placed on executive/attentional resources in various domains and contexts. This should be a source of comfort to researchers who make use of such effects to infer and study fundamental processes related to human cognition, since recent work on mind wandering would suggest that there may be significant effects of off-task rumination on performance in such tasks. Therefore mind wandering should not be thought of simply as an unavoidable hindrance, or even confound, in experimental psychology, but as a useful tool to be exploited in future work.

In summary, the present experiments represent the first empirical exploration of mind wandering in the context of reading aloud and color naming. It was found that mind wandering rates were quite high and negatively predicted RTs and errors across both of the experiments reported here. While this finding is in line with attentional resource accounts of mind wandering, the obvious lack of correspondence between performance deficits associated with mind wandering and variations in mind wandering across conditions in both tasks necessitates a reconsideration of the relationship between resource competition (between on and off-task thought) and primary task performance. Specifically, we argue that, at least in the kinds of tasks studied here, there is a point beyond which additional attentional resource investment affords little or no appreciable benefit to performance, and that consequently, individuals are able to make online adjustments to the distribution of attentional/executive resources that facilitates the pursuit of both internal and external goals.

### Conflict of interest statement

The authors declare that the research was conducted in the absence of any commercial or financial relationships that could be construed as a potential conflict of interest.

## Footnotes

^1^Although we report only the analyses from the five trials prior to the thought probes, we conducted identical analyses using only one, two, three, or four trials prior to the thought probes. The exact same pattern of data was obtained from these analyses as in the five-trials back analysis reported here.

^2^As in Experiment 1, although we report only the analyses from the five trials prior to the thought probes, we conducted identical analyses using only one, two, three, or four trials prior to the thought probes. The exact same pattern of RTs was obtained from these analyses as in the five-trials back analysis reported here.

^3^We remain deliberately agnostic as to whether this type of adjustment in the distribution of executive resources occurs implicitly or explicitly and suggest that it needs to be addressed in future work.
